# A pilot study of quantitative real-time polymerase chain reaction metastases detection on sentinel lymph nodes of oral cancer and literature review

**DOI:** 10.1590/1806-9282.2024S126

**Published:** 2024-06-07

**Authors:** Eduardo Vieira Couto, Fabio Lau, Fabio Portella Gazmenga, Daniel Texeira, Carmen Sílvia Bertuzzo, Carlos Takahiro Chone

**Affiliations:** 1 Universidade Estadual de Campinas, Department of Otolaryngology Head and Neck Surgery – São Paulo (SP), Brazil.; 2 Universidade Estadual de Campinas, Medical Sciences College, Department of Medical Genetics – São Paulo (SP), Brazil.

## INTRODUCTION

Head and neck squamous cell carcinoma (HNSCC) has a feature of lymphatic dissemination^1,2^. It represents a major prognostic factor^1,3–13^. However, the correct identification of metastatic deposits in lymph nodes (LNs) lacks effectiveness in the early stages of the disease^1^. Physical examination and imaging have proven unreliable, with false-positive and false-negative rates reaching 30%^14,15^.

Inaccurate diagnosis of LN metastases can lead to unnecessary up-front neck dissection and increased morbidity in cN0 patients. Conversely, intraoperative frozen section analysis misses small metastatic lesions and could jeopardize permanent sections^1^. A recent approach suggests limiting surgery to nodal staging using sentinel lymph node (SLN) biopsy^16^. This strategy aims to select only pN+ necks in patients for subsequent neck dissection^16^. If the SLN biopsy is negative, neck dissection can be avoided^9^.

Currently, there are different methods to analyze SLNs. A significant advantage of the frozen section is its intraoperative applicability, but sensitivity and negative predictive values range from 50 to 93%^16–20^ and 85.7 to 99%^18–20^, respectively. The method is subject to sampling errors, accuracy depends on the experience of the pathologist^21,22^, and material loss can lead to false-negative results^16^. Trivedi et al.^20^ demonstrated that the intraoperative analysis of SLNs failed to identify micrometastases and isolated tumor cells (ITC) in most patients. Although the clinical significance of ITC detection is controversial^20,23^, the presence of these smaller foci of metastasis is considered to be pathologic and led to neck dissection in the Sentinel European Node Trial^23^.

Even under microscopy, small tumor foci may not be detected, suggesting that 7–10% of pN0 patients have nodal recurrence even after elective neck dissection^22,24^. The current reference method is immunohistochemistry and step serial section^16,25^. Approximately 8–20% of patients with HNSCC have LN micrometastases on immunohistochemistry that are not detected by routine histopathological examination^7,26^. If immunohistochemistry confirms metastases not detected by hematoxylin-eosin, the patient may need a second surgical procedure^9^. However, the long time required prevents the use of this method for intraoperative diagnosis^16,26,27^.

The accuracy of the intraoperative diagnosis of neck involvement is the gap to be filled in evaluating SLNs. In this respect, molecular techniques for detecting LN metastases have been investigated. A promising alternative is reverse transcription-polymerase chain reaction (RT-PCR). This study aimed to observe our results in a pilot study of RT-PCR in SLN biopsy in our institution, to review the literature on molecular techniques using RT-PCR with a focus on the tumor markers for neck metastases from HNSCC and to estimate the time required in SLN biopsy with quantitative RT-PCR (qRT-PCR).

## METHODS

Three patients with cT1N0 of the lateral border of the tongue were consecutively enrolled in this study according to AJCC Eighth Edition for staging the primary lesions and neck. All were submitted to intraoral resection of the primary tumor with sentinel node biopsy. All participants were radiologically negative for lymphatic metastases by multi-slice computerized tomography (CT) scan with 128 detectors. All were submitted to two peri-tumorous injections of 0.2 MCI of fitato99m-TC 2 h before the surgery and lymphoscintigraphy 2 h after the injection. The activity of 25.6 MBq was injected along the submucosa of the normal mucous membrane surrounding the tumor in a volume of approximately 0.2 mL. Static images were accomplished in lateral and anteroposterior projections, and the radioactive LNs were marked in the skin. Lymphoscintigraphy and SPEC-CT were performed in all cases. The neck skin was marked accordingly, and a gamma probe was used to identify the sentinel LN intraoperatively. The handheld GP Neoprobe-1500 (Neoprobe Corp, Dublin, OH) identified the SLN in vivo and dissected and confirmed it ex vivo. Afterward, the remaining neck was re-evaluated for the absence of radioactivity. All LNs with radioactivity were dissected and considered SLN up to 10% of the first count. We obtained step serial sections at each 150 μm of the sentinel LN stained with hematoxylin-eosin and immunohistochemistry for cytokeratin AE-1/AE-3 in negative SLNB on HE.

Each SLN RNA was extracted from SLN biopsy samples to estimate the time required for molecular marker analysis in SLN biopsy by standard RT-PCR.

The Aurum™ Total RNA Mini Kit (Bio-Rad #732-6820) was used to extract three samples from different patients’ peripheral blood from three lymphocyte samples used as controls. The entire purification process of the samples, including DNase I digestion, was completed in 35 min.

The TaqMan MicroRNA Reverse Transcription kit (Life Technologies, New York, USA) synthesized complementary DNA from total RNA extracted from biological samples. The reactions were carried out at 16°C for 10 min, 42°C for 30 min, and 85°C for 5 min in an Eppendorf 22331 thermal cycler (Eppendorf, Hamburg, Germany).

Real-time qRT-PCR was performed using *EpCAM* gene expression reagent kit—amplicon length of 95 (Thermo Fisher number 4331182), *DSG3* gene expression reagent kit—amplicon length of 69 (Thermo Fisher number 4331182), and *HMBS* gene expression reagent kit—amplicon length of 125 (Thermo Fisher number 4331182). All kits use FAM™ (6-carboxyfluorescein) as the fluorophore.

Reactions were performed in triplicate in a 7500 real-time PCR system (Applied Biosystems, Thermo Fisher Scientific, Waltham, USA) with an initial incubation of 50°C for 2 min and 95°C for 10 min, and then 40 cycles of 95°C for 15 s and 60°C for 60 s. Relative quantification values were obtained by analyzing the results in the 7500 System SDS software (Applied Biosystems, Thermo Fisher Scientific, Waltham, USA) using the comparative CT method (ΔΔCT)^28^, considering the *HMBS* gene (former *PBGD*) as a reference.

We reviewed PubMed, Google Scholar, and UniGene (http://www.ncbi.nlm.nih.gov/) databases to identify the most relevant molecular markers for HNSCC metastasis in SLN biopsy.

The study was approved by the Research Ethics Committee of our institution (protocol number 24763919.9.0000.5404, approval number 4.070.277).

## RESULTS

The samples were assayed in triplicate to estimate the time spent on molecular analysis by RT-PCR, and the relative expression values are summarized in Table 1.

**Table 1:** Expression of *PVA* and *TACSTD1* genes related to squamous cell carcinoma metastases in sentinel lymph node biopsy samples relative to the *PBGD* control gene in lymphocytes from controls.

Samples	Mean expression — *PVA (DSG3)*	Repetition A	Repetition B	Repetition C	Mean expression —*TACSTD1/EpCAM*	Repetition A	Repetition B	Repetition C
SLN 1	37.666796	43.097121	32.116560	37.786709	45.751501	47.457656	41.453422	48.343427
SLN 2	0.0000036	0.000001	0.000003	0.000007	0.006088	0.005421	0.003211	0.009632
SLN 3	12.700829	12.544412	10.342264	15.215812	22.378324	22.580421	23.786763	20.767790
C 1	-2.168592	-2.167878	-2.169900	-2.167998	-3.184274	-2.191000	-3.145231	-4.216591
C 2	-2.174203	-2.171666	-2.180988	-2.169955	-3.965620	-3.345600	-3.548941	-5.002321
C 3	-2.168125	-2.166499	-2.166999	-2.170877	-2.322124	-2.456452	-2.183421	-2.326501

SLN: sentinel lymph node; C: control.

**Figure 1:**
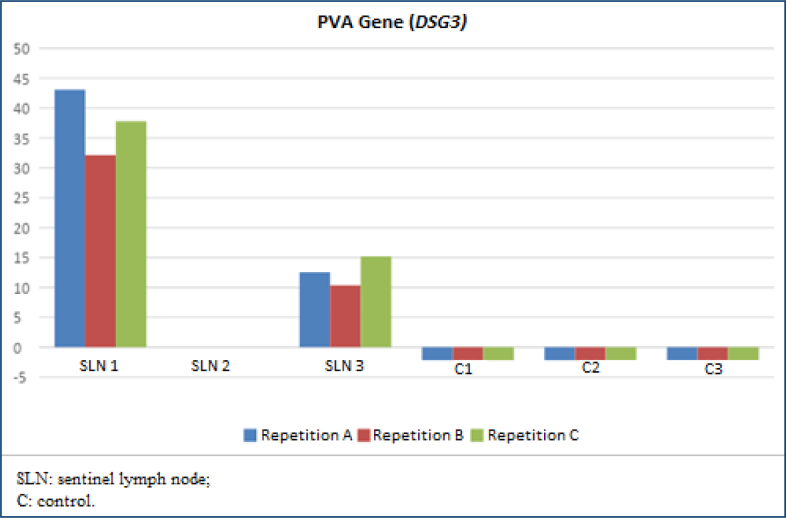
Gene expression detected by real-time polymerase chain reaction of the *PVA* gene (also called *DSG3*) in three sentinel lymph node biopsies and normalization as controls was performed with the expression of the *PBGD* gene (also called *HMBS*) in three lymphocyte samples from the controls. All models were made in triplicates (blue, red, and green).

**Figure 2:**
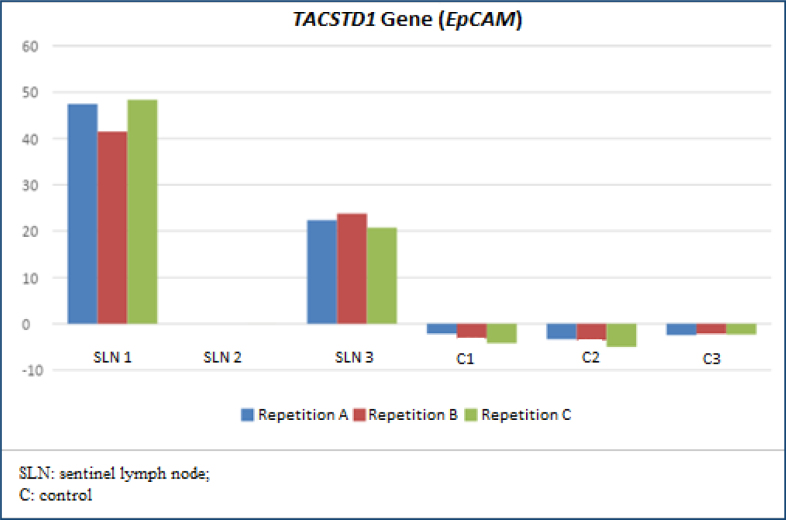
Gene expression detected by real-time polymerase chain reaction of the *TACSTD1* gene (also called *EpCAM*) in three sentinel lymph node biopsies and normalization as controls was performed with the expression of the *PBGD* gene (also called *HMBS*) in three lymphocyte samples from the control. All models were made in triplicates (blue, red, and green).

Each assay step took 35 min for RNA extraction, 45 min for conversion of RNA to DNA, and 62 min for qPCR. The total laboratory procedure time was 2 h and 22 min, withholding the time spent on pipetting and transporting samples. SLN 2 was negative for metastasis, while SLN 1 and 3 were positive for metastases because they expressed proteins above normal. SLN 1 and 3 were also histopathological positive for SCC metastasis in hematoxylin-eosin, step serial sectioning analysis. This sample's relative gene expression values observed a difference between biopsies and relative to lymphocyte controls expression too but with a minimal expression of these last ones (Figures 1 and 2 and Table 2).

**Table 2:** Analysis of molecular markers by real-time polymerase chain reaction.

References	Marker	Sensitivity	Specificity	Accuracy
Ferris et al.^29^	PVA	100%	100%	100%
	TACSTD1	98.3%	94.4%	96.2%
	PTHrP	93.8%	98.8%	96.3%
	SCCA 1/2	99.8%	98.5%	99.1%
Ferris et al.^17^	PVA	92%	98%	98%
	TACSTD1	70%	99%	95%
	PTHrP	60%	98%	95%
Solassol et al.^27^	PVA	100%	100%	100%
	SCCA	100%	93.7%	95.4%
	CK17	100%	81.2%	86.3%
Garrel et al.^16^	CK17	100%	100%	100%
Shores et al.^25^	CK14	100%	96%	**

** Data not available in the study.

*PVA:* pemphigus vulgaris antigen; *TACSTD1:* tumor-associated calcium signal transducer 1; *SCCA:* squamous cell carcinoma antigen; *E48:* squamous cell carcinoma specific antigen E48 (Ly-6D); *PTHrP:* parathyroid hormone-related protein; CK: cytokeratin.

Our review identified 10 potential molecular markers in the search for cervical metastases from HNSCC: pemphigus vulgaris antigen (*PVA*), *TACSTD1*, squamous cell carcinoma antigen (*SCCA*), *E48*, parathyroid hormone-related protein (*PTHrP*), and cytokeratins (*CK13*, *CK14*, *CK17*, *CK19*, and *CK20*).

Using real-time qRT-PCR, Solassol et al.^27^ found no significant difference in the *PVA*, *SCCA*, and *CK17*. However, there was a significant substantial difference in the levels of the three markers between positive and negative LNs (Table 2). Cutoff values were calculated to maximize sensitivity. For a 27.3% prevalence of SLN invasion, the positive predictive values of *CK17*, *SCCA*, and *PVA* were 79.3, 91.2, and 100%, respectively^27^.

Among 40 potential molecular markers, Ferris et al.^29^ identified *PVA*, *TACSTD1*, *PTHrP*, and *SCCA 1* and *2* as possible detectors of LN metastases from HNSCC. In a later study, the authors compared the results obtained by qRT-PCR for *PVA*, *PTHrP*, and *TACSTD1* with pathological analysis performed in 35 min^17^.

Using RT-PCR, Nieuwenhuis et al.^8^ showed *E48*-positive signals in LNs in 22% of pN0 patients and 56% of pN+ patients. In the same study, of 15 patients with *E48*-positive LNs, seven were upstaged regarding N-stage (N0 to N1 or N1/2a to N2b)^8^.

Garrel et al.^16^ assessed the accuracy of qRT-PCR targeting *CK5*, *CK14*, and *CK17* in HNSCC SLNs. The mean duration of qRT-PCR was 180 min. The area under the curve was 87.1% for *CK5*, 82.8% for *CK14*, and 100% for *CK17*, but *CK17* performed better based on the cutoff value determined by the authors (Table 1)^16^. Shores et al.^25^ showed 100% sensitivity and 96% specificity for *CK14* by qRT-PCR, with five possible false positives of a total of 138 negative LNs.

Hamakawa et al.^30^ investigated *CK13*, *CK19*, and *CK20* by RT-PCR in primary tumors and cervical LNs of patients with oral SCC. *CK19* was detected in 40% of control LNs, whereas *CK13* and *CK20* were undetectable. *CK13* and *CK19* were expressed in all primary tumors, whereas *CK20* was present in only 40%. Of 13 positive LNs, all expressed *CK13*, one did not express *CK19*, and six had undetectable *CK20* levels. Of 166 negative LNs, *CK13* was expressed in 14.4%, *CK19* in 54.4%, and *CK20* in 3.0%^30^.

## DISCUSSION

In cN0 patients, SLN biopsy has proven a valuable tool in selecting pN+ in cN0 patients for neck dissection and avoiding surgery for those with negative SLNs, associated with decreasing morbidity in pN0 patients compared with upfront neck dissection. However, this strategy has a sensitivity of 50–85%^16,17^, with a negative predictive value of 97%^31^. However, for 29% of patients^31^, the SLN could come positive, and a second-stage neck dissection would be necessary. Intraoperative diagnosis of SLN with frozen section has low sensitivity compared with permanent sectioning and demands special microtomes. To avoid a second-stage approach intra-operatively, molecular markers are potential tools for evaluating SLNs in patients with HNSCC if executed within an acceptable time with good reliability.

*E48* is an antigen expressed in normal, malignant, and transitional squamous epithelial cells^8^. Nieuwenhuis et al.^32^ investigated the diagnosis of micrometastases in LN aspirates. It showed real-time qRT-PCR and greater sensitivity in the cytological examination. Subsequently, the same authors investigated *E48* as a potential marker for detecting HNSCC in LN samples by RT-PCR and compared the results with histopathological examination. *E48* was detected in 22% of pN0 patients and 56% of pN+ patients in at least one histologically tumor-negative LN. There was nodal upstaging in 7 of 15 patients with *E48*-positive LNs^8^.

Ferris et al.^17^ compared the qRT-PCR results obtained for *PVA*, *PTHrP*, and *TACSTD1* with pathological examination using hematoxylin-eosin and immunohistochemistry staining. Despite demonstrating *PVA's* ability to detect micrometastases in LNs, the same authors pointed out possible limitations^17,21^. In 103 LNs, of which 43 were positive, the assay for *PVA* and *TACSTD1* failed to identify three metastatic LNs with 5% or fewer tumor cells on only one section. The authors’ hypothesis for these false-negative LNs was sampling error^17^.

Solassol et al.^27^ evaluated the applicability and accuracy of real-time RT-PCR with *PVA*, *SCCA*, and *CK17* (Table 2) by comparing it with histopathological examination of 78 SLNs obtained from 22 patients with HNSCC and 11 control LNs from patients without cancer. *PVA* was the only marker distinguishing LNs with micrometastases from negative LNs. No false-negative cases were observed for *PVA*, whereas one and three patients were misclassified with *SCCA* and *CK17*, respectively. None of the markers differentiated ITC from negative LNs, which the authors attributed to a possible sampling error^27^.

Hamakawa et al.^6^ investigated *SCCA* gene expression using RT-PCR to detect cervical micrometastases from HNSCC by comparison with histopathological examination of 212 LNs obtained from 21 patients. Of 198 histologically negative LNs, *SCCA* mRNA was positive in 37 (18.7%) and upstaged the N-stage of 14 patients^6^. In a later study, Hamakawa et al.^33^ evaluated 10 patients with cN0 oral SCC who had undergone SLN biopsy. One-half of each LN was subjected to frozen section analysis, while the other half was subjected to qRT-PCR for *SCCA* quantification. The method was performed manually within 2 h and 30 min, and no gene amplification of *SCCA* was observed in negative control LNs, but the automated process could speed up the analysis. Histopathological evaluation detected micrometastases in two LNs from different patients. *SCCA* was positive in these two LNs and staged as pN+. Moreover, even 2 h and 30 min is still faster than conventional histopathological evaluation with hematoxylin-eosin, step serial section, and immunohistochemistry as standard protocol for assessing SLN.

Shores et al.^25^ investigated the use of *CK14* qRT-PCR to detect occult metastases in 153 cervical LNs from 13 patients with HNSCC. One portion of each LN was subjected to histopathological examination, while the rest was subjected to RT-PCR. All histopathological positive LNs expressed *CK14*. The authors established an arbitrary cutoff value for *CK14* detection to avoid false-positive results. Thus, *CK14* had a sensitivity of 100% and a specificity of 96%, with five possible false positives. However, sampling error may have occurred, and the study methodology could not confirm conflicting results between the two analyses^25^.

Garrel et al.^16^ assessed the accuracy of qRT-PCR in staging SLNs by targeting *CK5*, *CK14*, and *CK17* and using immunohistochemistry as the reference test. The mean duration of qRT-PCR was 180 min. There was no significant difference in the levels of the three markers between controls and negative LNs. *CK17* and *CK14* showed a significant difference in positive LNs compared with negative LNs. *CK5* showed no significant difference between the groups. *CK17* performed better, with 100% sensitivity and specificity, based on the cutoff value determined by the authors. The positive and negative predictive values were 100% for a 41.18% prevalence of SLN invasion. *CK17* failed to detect two micrometastases in two patients, but its staging accuracy was not compromised due to the detection of metastases in other SLNs^16^.

Tao et al.^10^ evaluated the presence of occult micrometastases in 1,328 LNs from 31 patients with HNSCC by real-time *CK19* qRT-PCR and compared the results with histopathological examination. The LN metastatic rates determined by histopathology and RT-PCR were 16.3 and 36.0%, respectively. The N-stage of 42% of patients would have changed if the molecular analysis had been considered. Furthermore, *CK19* expression levels were significantly higher in positive than negative LNs^10^.

In addition to *SCCA*, Hamakawa et al.^30^ investigated the expression of *CK13*, *CK19*, and *CK20* by RT-PCR in primary tumors and neck LNs of patients with oral SCC. They concluded that *CK20* has less value in diagnosing cervical metastasis due to the low detection rate in primary tumors and positive LNs. *CK19* has a high detection rate in negative cervical LNs, possibly due to illegitimate gene expression of leukocytes, *CK19* pseudogene of tissue, or gene expression from ectopic salivary glands. *CK13* would change the N-stage of 100% of pN0 patients^30^.

The few studies assessing molecular markers for detecting LN metastasis in HNSCC used different methodologies with small sample sizes and have sometimes been conducted in the context of SLN evaluation. A limitation of this method is the possibility of false positives due to the presence of ectopic salivary glands^6,16,21,26,30^. Hamakawa et al.^6,33^ acknowledge that normal salivary glands express *SCCA* in a small volume, which is insufficient to achieve a certain cutoff value. Also, none of the ectopic salivary glands in cervical LNs expressed *SCCA* ^6^. However, the same authors showed *CK13* and *CK19* expression in salivary glands^30^. Sampling error has also been considered a limitation in several studies^16,17,26^.

Another crucial point is the time spent on genetic testing. Based on the results of our equipment, it took us approximately 2 h and 30 min to run the assay manually. The shorter the assay duration, the greater the benefit in the intraoperative study for LN metastases through SLNs. Ferris et al.^17^ described using an automated system to analyze *PVA* and *TACSTD1* genes, in which the assay was completed in approximately 35 min. We contacted the company's representative and were informed that the Ferris et al.^17^ cartridges were customized and compounded. Currently, only closed, pre-loaded diagnostic cartridges are available for purchase. For the manufacture of personalized cartridges, additional time would have to be added for production and import.

Although the intraoperative frozen section has been used to evaluate SLN, its sensitivity reported is variable, the accuracy depends on the experience of the pathologist^21,22^, and the loss of material can lead to false-negative results^16^. If validated, qRT-PCR is potentially more sensitive than histopathology as it can be used to sample the entire LN or a significant portion of it. Furthermore, qRT-PCR is an objective method that does not depend on the examiner's interpretation and removes any doubt of potential human error with the sample processing. Finally, SLN samples can be processed to permit both qRT-PCR and routine pathological evaluation in parallel on adjacent tissue sections.

The ability to stage the cN0 neck has great clinical application accurately and rapidly to avoid the morbidity associated with open neck dissection in pN- or a second surgery in pN+ patients. These studies show that molecular tumor markers can be used with qRT-PCR to accurately predict nodal metastases in SLN samples in the intraoperative time frame during the procedure to remove the primary tumor or the closure of incisions. Although they are pilot studies, such an analysis is innovative. All showed excellent sensitivity, specificity, and accuracy rates of molecular analysis compared with conventional histopathological and immunohistochemical analysis, with variations of 60–100%, 81.2–100%, and 86.3–100%, respectively.

The lack of accurate diagnosis in the intraoperative frame time of SLNs is a topic of intense interest in the head and neck oncologic community. A delayed pN diagnosis compels pN+ patients to undergo further additional surgery with an increased risk of postoperative complications, damaged function, and worse outcomes. The advent of molecular markers and the development of rapid and precise molecular techniques can fill the gap in evaluating SLNs for identifying and treating LN metastases in HNSCC. If the molecular marker confirms metastases in SLN, the patient will not need a second surgical procedure, and the neck dissection could be done in the same procedure avoiding time delay to adjuvant treatment if required and second hospitalization, which could be a problem in the context of reschedule of another surgery in the same patient.

## CONCLUSION

The estimated time for molecular analysis of an SLN biopsy sample by qRT-PCR was approximately 2 h and 30 min. Despite the limitations and few studies, molecular analysis for the diagnosis of lymphatic metastasis in SLN of oral cancer is a promising tool that can help guide surgeons’ decision-making in the intraoperative diagnosis of SCC metastasis in SLNs.
